# Inequalities in extending working lives beyond age 60 in Canada, Denmark, Sweden and England—By gender, level of education and health

**DOI:** 10.1371/journal.pone.0234900

**Published:** 2020-08-17

**Authors:** Ashley McAllister, Theo Bodin, Henrik Brønnum-Hansen, Lisa Harber-Aschan, Ben Barr, Lee Bentley, Qing Liao, Natasja Koitzsch Jensen, Ingelise Andersen, Wen-Hao Chen, Karsten Thielen, Cameron Mustard, Finn Diderichsen, Margaret Whitehead, Bo Burström

**Affiliations:** 1 Department of Global Public Health, Karolinska Institutet, Stockholm, Sweden; 2 Melbourne School of Population and Global Health, The University of Melbourne, Melbourne, Australia; 3 Center for Occupational and Environmental Medicine, Stockholm County Council, Stockholm, Sweden; 4 Institute of Environmental Medicine, Karolinska Institutet, Stockholm, Sweden; 5 Department of Public Health, University of Copenhagen, Copenhagen, Denmark; 6 Department of Public Health and Policy, University of Liverpool, Liverpool, United Kingdom; 7 Institute for Work and Health, Toronto, Canada; 8 Social Analysis and Modelling Division, Statistics Canada, Ottawa, Canada; 9 Unit of Social Medicine, Frederiksberg Hospital, Copenhagen, Denmark; 10 Dalla Lana School of Public Health, University of Toronto, Toronto, Canada; Tulane University, UNITED STATES

## Abstract

**Background:**

Keeping older workers in employment is critical for societies facing the challenge of an ageing population. This study examined the association between types of health conditions and differentials in the probability of employment by level of education among men and women between 60–69 years of age in Canada, Denmark, Sweden and England.

**Methods:**

Data were drawn from the Canadian Community Health Survey, Survey of Health, Ageing and Retirement in Europe and English Longitudinal Study of Ageing. We combined country data, applied logistic regression, adjusted for educational level, and stratified the analysis by sex to calculate the odds ratio (OR) of employment (>15 hours work per week) for persons with physical health conditions, mental health conditions (depression) and physical-mental health comorbidity.

**Results:**

The odds of employment among men and women with physical-mental health comorbidity were lower compared to those with no/other conditions (men: OR 0.32, 95% CI: 0.25–0.42, women: OR 0.38 95% CI: 0.30–0.48). Women with low education had lower odds of employment compared to their counterparts with high education (OR 0.66, 95% CI: 0.57–0.76). The odds of employment at older ages was lower in Canada, Denmark and England compared with Sweden (e.g. English men: OR 0.48 95% CI 0.40–0.58; English women OR 0.33 95% CI 0.27–0.41).

**Conclusions:**

The odds of employment beyond age 60 is lower for groups with low education, particularly women, and those with physical-mental health co-morbidities. As such, policies to extend working lives should not be ‘one size fits all’ but instead consider subgroups, in particular, these groups that we have shown to be most vulnerable on the labour market.

## Introduction

Keeping older workers in employment is critical for societies facing the challenge of an ageing population. In the European Union (EU), the dependency ratio (i.e. the number of people of working age (15–64 years of age) for every person above 65 years of age), is expected to halve from 4:1 to 2:1 by 2060 [1] and this decline in the proportion of people of working age is expected to slow economic growth [2]. However, extending working lives also highlights the issue of health and social inequalities, as the possibilities to work beyond or even until the current pension age vary between men and women, between those with health problems and those without, and between higher and lower socioeconomic groups. The number of people with chronic health conditions is rising in tandem with increasing life expectancy in almost all developed societies [3].

An early exit from the labour market is a complicated process taking place over an extended period and is dependent on several factors at a personal, family and societal level, where health is one of the most important factors [4]. Workers with chronic health conditions have an increased risk of early retirement [5]. However, some persons with health conditions continue to work into older ages [6] and under the right circumstances, even more could remain in employment longer. However, a gap exists in the literature as to whether the probability of employment is more likely with certain chronic health conditions compared to others.

Gender is also another factor that could influence extending working lives. In most cases, employment rates are lower for women than for men [7]. Compared to men, women are also more likely to work part-time, and take more days off from work to care for children and family members [8–10]. Women are also more likely to be absent from work due to sickness, especially due to mental health issues [11]. For the individual, reduced employment during prime working years has spillover effects into later life, including limited career options, lower wages and subsequently lower pensions [12]. Hence, older women face the dual challenge of underemployment and higher prevalence of chronic health conditions. However, little is known about the differences among men and women in employment related to health status across different contexts for older workers.

Finally, socioeconomic status (SES) is also vital to extending working life. Some evidence shows that working life differences exist between workers with low and high SES [13–15]. Beyond that, life expectancy inequalities also exist between these groups [16]. One systematic review also shows that those in lower SES groups (e.g. labourers, machine operators) are at elevated risk of suicide compared with other occupational groups, particularly in high SES groups [17]. Overall, lower skilled workers, have shorter life expectancy, earlier onset of chronic illness [18] and are more likely to suffer co-morbidities as they age [19]. Given the sparse literature on employment among older workers, more is needed on SES inequalities that may exist among this group.

Previous comparative studies focused on health and employment of the general working population illustrate that it is important to consider institutional context [20–23]. In this study, we compare Canada, Denmark, Sweden and England. This study is part of a larger project entitled “Tackling Health Inequalities and Extending Working Lives” (THRIVE) which is a consortium involving researchers from Canada, Denmark, Sweden and England. McAllister et al. [6] outline the THRIVE theoretical framework, provide a rationale for comparing these countries and an overview of the four countries. Table 1 provides a brief summary of the four countries. Overall, the THRIVE project hypothesises that policies aimed at increasing employment among the general working age population (e.g. policies in Nordic welfare regimes, including Denmark and Sweden) would facilitate higher employment rates also among older workers compared to more punitive policies (e.g. those in market-oriented welfare regimes, including Canada and UK). Bambra and Eikemo [21] argue that social protection (e.g. generosity of welfare benefits) plays an important role in the relationship between unemployment and health (e.g. more generous benefits tend to lead to better health outcomes) but that this varies by welfare regime. McAllister, et al. [24] argue that in addition to social protection, employment protection legislation and active labour market policies also play a role in the relationship between employment and health. However, little analysis is available on the relationship between employment and health for older workers in a comparative context focusing on population subgroups [21], even less on specific health conditions (most studies use self-rated health or limiting longstanding illness rather than specific diagnoses). In this paper, we address this gap.

**Table 1:** Brief overview of comparison countries.

	Canada	England	Denmark	Sweden
Welfare Regime	Market oriented	Market oriented	Nordic	Nordic
Population	36 million	66 million	5.8 million	10million
Pension age (not mandatory)	65 years	65 years	65 years	N/A
Disability benefits	Less generous and moderate eligibility	Less generous and strict eligibility	Generous but strict eligibility	Generous but strict eligibility
Employment protection	Moderate	Weak	Flexicurity approach	Strong
Spending on active labour market policies	Low	Low	High	High

Data from table taken from [24] and [25].

Most studies do not examine health and the probability of employment past the age of 65 years, and few are comparative (with some exceptions [6, 20]). We contribute to the literature by examining the association between three different categories of health conditions and employment among men and women aged 60–69 years in Canada, Denmark, Sweden and England and whether the association varies across the four countries. This study builds on a previous theoretical and descriptive paper from the THRIVE project [6]. This current study complements the findings of the McAllister et al. [6] and contributes to the literature by presenting detailed comparative regression analysis on how physical and/or mental health conditions are associated with the likelihood of employment among older workers; by combining data from the different countries into one dataset. Combining data allows for a more rigorous statistical comparison between different countries in the same analysis. To the best of our knowledge, we found no other studies that combine country data in this way for comparative analysis of employment among older workers. Our paper provides evidence on inequalities that need tackling to extend working lives.

## Material and methods

### Study population

We use a comparative cross-sectional study design drawing on nationally representative cross-sectional survey data. We use the 2013 (Wave 5) Survey of Health, Ageing and Retirement in Europe (SHARE) [26] for Denmark and Sweden, pooled 2012–13 years from the English Longitudinal Study of Ageing (ELSA) [27] for England, and pooled 2012–14 years Canadian Community Health Survey (CCHS) for Canada [28]. We restrict our sample to individuals aged 60–69 years.

### Measures

#### The outcome of interest

Employment was our outcome of interest, defined as working more than 15 hours per week in the survey week. This cut-off was selected in order to capture part-time workers who are common among this age-group, whilst still making a substantial contribution to the labour market than the common cut-off of 1 hour or more per week [6].

#### Exposures

We selected cardiovascular disease, diabetes, depression, musculoskeletal and respiratory disease. These five conditions represent major causes of disability pension and burden of disease in the four countries [29–33]. We further categorised these conditions into the following:

#### Physical health condition

We defined respondents as having a physical health condition if they reported having one or more of the following conditions: cardiovascular disease, diabetes, musculoskeletal and respiratory disease, measured by asking respondents to self-report whether they have the specific condition or not.

### Mental health condition

#### Depression

We defined respondents as having a mental health condition based on whether they reported symptoms or a diagnosis of depression. In SHARE this was based on a score of greater than or equal to 4 on the validated EURO-D scale [34]. In ELSA this was based on a score of greater than or equal to 3 on the validated Center for Epidemiological Studies Depression (CES-D) [35, 36]. In the CCHS this was based on whether the respondent reported that they had been diagnosed with depression. We recognise that depression is only one mental health condition, but depression is the leading cause of disability worldwide and has a significant impact on working life [37].

#### Physical-mental health comorbidity

We defined respondents as having at least one of the selected physical health conditions **AND** our chosen mental health condition–depression. The intersection between physical and mental health conditions has been shown to be particularly burdensome, as evidence suggests that this acts through bidirectional mechanisms to further impair health and occupational functioning [38].

#### No/other conditions

*This* category included persons with no health conditions AND persons with health conditions other than the five selected in this paper.

### Socioeconomic status (SES)

We used educational level as an indicator for SES. Two educational levels were measured using the International Standard Classification of Education (ISCED) categories and divided into two subcategories: Low (0, 1, 2, 3, 4) and High (5 and 6). SHARE uses the ISCED categories to measure education. In ELSA and the CCHS, education is measured by asking respondents their highest qualification. We coded the response into a 2-category response which can be matched against the classification used in ISCED.

### Sex and age

We focus on men and women aged 60–69 years.

See Table 2 for comparison of variable definitions across the datasets.

**Table 2:** Summary of definition of variables by dataset.

Variable definition	Canadian Community Health Survey (CCHS)	Survey of Health, Ageing and Retirement in Europe (SHARE)	English Longitudinal Survey on Ageing (ELSA)
Employment	Indicate the number of hours worked in the previous week	Indicate total hours usually working per week	Indicate the number of hours worked in the previous week
Physical condition	Have you been diagnosed with (specific condition)?	Combination of specific conditions and specific drug use	Has a doctor told you that you had (insert condition) on this card?
Depression	Have you been diagnosed with depression?	EURO-D ≥ 4	Centre for Epidemiological Studies Depression Scale (CES-D) ≥ 4
Educational level	What is the certificate, diploma or degree that [respondent’s name] has completed?	International Standard Classification of Education (ISCED) categories and divided into two subcategories: Low (0,1,2,3,4) and High (5 and 6).	Derived variable (qual2): higher than o-level, o-level or below.

### Ethical approval

We obtained ethical approval from each participating country. For Canadian data, the study methods were reviewed and approved by Statistics Canada officials at the University of Toronto Research Data Centre. In Denmark, the National Committee on Health Research Ethics does not require ethical approval to use SHARE Wave 5 as it is solely based on survey data and does not include any samples of biological materials from humans (http://en.nvk.dk/how-to-notify/what-to-notify). However, the SHARE project is submitted to continuous ethics reviews. From wave 4 and onwards SHARE has received ethical approval from the Ethics Council of the Max Planck Society. The last ethics approval was granted on 4 March 2016 (http://www.share-project.org/organisation/dates-facts.html). For Swedish data, the Swedish team received ethical approval to use the SHARE data from the Regional Ethical Review Board of Stockholm (Dnr 2016/1353-31/5). Approval to use the ELSA data is provided by the UK Data Service under an End User License agreement. All respondents have given written and informed consent. Ethical approval for the original study was provided by the National Research and Ethics Committee (MREC/01/2/91). For all surveys, participants gave full informed written consent to participate in the surveys.

### Statistical methods

The analysis was performed by aggregated data collected from each country. Thus, the micro data were combined and then analysed using logistic regression models to account for country effects and to investigate cross-national differences. Due to different sizes of the surveys from the four countries the analyses were weighted accordingly. Models stratified by sex estimated the odds ratio (OR) of being in employment. Low power due to small survey samples (except for Canada) limited the possibility to include confounders and interaction terms in the model. Thus, only age (two categories), education (two categories), and country were included in the fully adjusted model (Model III).

## Results

Table 3 shows the characteristics of the study population. Canada contributed with 36,195 individuals (86% of the total sample) followed by England 2,789 (7%), Sweden 1,821 (4%) and Denmark 1,389 (3%). Overall, the proportion of men and women and the two age groups were similar across the four countries. The proportion of persons working > 15 hours per week differed across the countries ranging from 26.3% in England to 40.8% in Sweden. Regarding high education, Canada had the highest proportion (57.3%), while England had the lowest proportion (30.3%). The countries also varied in the proportion of persons with the different health categories, with proportions being similar between Denmark and Sweden, and to a lesser extent, between Canada and England. The largest variation was with physical-mental health comorbidity ranging from 9.5% in Canada to 23.0% in Sweden.

**Table 3:** Individual characteristics, work characteristics and health conditions among persons aged 60–69 years in CAN, DK, SE and ENG in 2012–14.

	CAN^a^	DK^b^	SE^b^	ENG^c^
	n (%)	n (%)	n (%)	n (%)
**Total**	36 195 (100)	1389 (100)	1 821 (100)	2 789 (100)
**Men**	15 866 (49.1)	667 (48.0)	857 (47.1)	1 354 (48.6)
**Women**	16 489 (50.9)	722 (52.0)	964 (52.9)	1 435 (51.4)
**Age (years)**				
60–64	17 849 (55.2)	671 (48.3)	808 (44.4)	1 458 (52.3)
65–69	14 507 (44.8)	718 (51.7)	1 013 (55.6)	1 331 (47.7)
**Education**				
Low	13 820 (42.7)	749 (53.9)	1 177 (64.6)	1 944 (69.7)
High	18 535 (57.3)	624 (44.9)	598 (32.8)	845 (30.3)
**Employment**				
Employed (> 15 hours/week)	12 104 (37.4)	462 (33.3)	743 (40.8)	734 (26.3)
**Health condition**				
No/other conditions	9 182 (28.4)	284 (20.5)	414 (22.7)	743 (26.7)
Physical only	19 518 (60.3)	698 (50.3)	840 (46.1)	1 544 (55.4)
Depression only	572 (1.8)	94 (6.8)	148 (8.1)	86 (3.1)
P-M Comorbidity	3 082 (9.5)	313 (22.5)	419 (23.0)	415 (14.9)

^a^ Based on 2012–14 CCHS data

^b^ Based on Wave 5 2013 SHARE data

^c^ Based on 2012–13 ELSA data

Note: CAN–Canada; DK–Denmark; SE–Sweden; ENG–England.

The results for the association between the three health categories and the probability of employment stratified by sex are presented in Table 4. Odds ratios of being in employment are presented for the three health categories relative to the no/other conditions category, separately for men and women in three models, using combined data from all four countries. The analytic sample for the four countries combined was 42,154.

**Table 4:** Association between health conditions and likelihood of employment by gender in combined data from CAN, DK, SE and England for persons aged 60–69 years OR (95% CI) (n = 42,154).

	MEN			WOMEN		
	Model I (Crude)	Model II (adjusted for age & education)	Model III (adjusted Model II + country)	Model I (Crude)	Model II (adjusted for age & education)	Model III (adjusted Model II + country)
No/other conditions^a^	1	1	1	1	1	1
Physical only	0.59 (0.52–0.68)	0.66 (0.57–0.76)	0.65 (0.57–0.75)	0.54 (0.47–0.62)	0.58 (0.50–0.68)	0.58 (0.50–0.67)
Depression only	0.80 (0.51–1.24)	0.74 (0.45–1.22)	0.74 (0.45–1.21)	0.98 (0.69–1.40)	0.91 (0.63–1.30)	0.90 (0.61–1.32)
P-D comorbidity	0.33 (0.26–0.42)	0.31 (0.24–0.40)	0.32 (0.25–0.42)	0.44 (0.35–0.55)	0.39 (0.31–0.50)	0.38 (0.30–0.48)
65–69 years^b^		0.20 (0.18–0.23)	0.20 (0.17–0.22)		0.16 (0.14–0.18)	0.14 (0.12–0.16)
Low Education^c^		0.90 (0.79–1.02)	0.88 (0.77–1.00)		0.64 (0.56–0.73)	0.66 (0.57–0.76)
Country						
SE			1			1
DK			0.70 (0.56–0.87)			0.51 (0.41–0.65)
CAN			0.70 (0.60–0.82)			0.54 (0.46–0.63)
ENG			0.48 (0.40–0.58)			0.33 (0.27–0.41)

Note: Odds is the probability of having at least 15 hours work per week.

^a^ The reference category: no/other conditions—includes those that reported no condition and those that reported health conditions other than the five specific conditions of interest

^b^ The reference category: 60–64 years

^c^ The reference category: high education

Note: CAN–Canada; DK–Denmark; SE–Sweden; ENG–England.

### Odds of employment among men

Men with physical-mental health comorbidity had substantially lower odds of employment compared to the odds of employment for the no/other conditions (OR 0.33, 95% CI: 0.26–0.42). These crude associations held after adjusting for age and education (Model II) and country (Model III). Men with physical health conditions had lower odds of employment compared to no/other conditions (OR 0.65, 95% CI: 0.57–0.75). Men who had depression only had lower odds of employment compared to no/other conditions, but this difference was not statistically significant (OR 0.74, 95% CI: 0.45–1.21). Men with low education had lower odds of employment compared to men with high education (OR 0.88, 95% CI: 0.77–1.00). Men aged 65–69 years had substantially lower odds of employment compared to men aged 60–64 years (OR 0.20, 95% CI: 0.17–0.22).

### Odds of employment among women

Women with physical-mental health comorbidity had lower odds of employment than no/other conditions (OR 0.44, 95% CI: 0.35–0.55). This strength of the crude associations increased after adjusting for age and education and country. Women who had depression only had lower odds of employment compared to no/other conditions, but this difference was not statistically significant (OR 0.90, 95% CI: 0.61–1.32). Women with low education had lower odds of employment than women with high education (OR 0.66, 95% CI: 0.57–0.76). Like men, older women (aged 65–69 years) had substantially lower odds of employment compared to women aged 60–64 years (OR 0.14, 95% CI: 0.12–0.16).

### Odds of employment by country

Of the four countries, the prevalence of employment was highest for Sweden which therefore served as the reference group in the logistic regression analysis. Relative to Sweden, the odds of employment were the lowest for England (OR 0.4 for English men compared to Swedish men, and OR 0.33 for English women compared to Swedish women). For men, the odds of employment were similar between Canada and Denmark (both OR 0.70). Women in Canada, Denmark and England were substantially less likely to be employed than women in Sweden.

## Discussion

We found inequalities in the odds of employment among persons aged 60–69 years, between men and women, by level of education and by health condition; and differences between countries. To a large extent these inequalities in the odds of employment reflect inequalities in the health conditions, but the difference in country fixed effects could also indicate that the policy environment, cultures, values or the way the chosen health conditions are diagnosed in each country may have an impact on the odds of employment in this age group.

### Health inequalities

Our results align with current evidence that the chances of employment are lower among those with health conditions [23, 38, 39] compared to those without health conditions. However, our study nuances the argument showing that inequalities exist *between* different health conditions. For example, men and women with physical-mental health comorbidity had substantially lower odds of employment relative to the no/other conditions category and compared to men and women with only depression or only the physical health conditions. Our data also show that the comorbidity group is several times larger than the “depression only” group in all countries. Such information is important for policymakers and employers, as our findings suggest that the retention of older workers in many cases would require workplace adaptations targeting both physical and mental health, as previous studies on determinants for staying at work suggest [40].

For people with depression only, the odds of employment were only slightly lower than the no/other conditions and was not statistically significant, suggesting that the presence of depression alone had a relatively small effect on respondents’ probability of employment at this age. One explanation could be that the numbers in our datasets for persons with self-reported depression are low compared to the prevalence of depression in all four countries. Some evidence suggests that depression among older persons is under-recognised and under-treated [41]. As such, some of those in the ‘other’ category could have unreported or undiagnosed symptoms of depression. Another explanation could be that given there is a strong association between chronic physical diseases and the onset of depression [41], it may be more likely among those older than 60 years to have depression with a physical health condition than only depression [42].

### Socio-economic inequalities

We found a strong association between low odds of employment among low-educated women compared to high educated women. Such results suggest that social inequalities may also exist in extending working lives. Our results support the literature on the general working age population that employment is more likely among those with high education [13–15, 43]. One cohort study focusing on older workers [20] found that workers with low SES were more likely to exit employment due to health reasons compared to those with high SES. More evidence is needed before conclusions about push and pull factors related to SES and extending working lives can be made.

### Differences between countries

Our results also showed a different cross-national pattern of employment by health category than we hypothesized. After adjusting for health condition, age and education, older women in Denmark, Canada and England had lower odds of employment than women in Sweden (similar observation for men, although differences were smaller). We hypothesised that results would be similar for Denmark and Sweden–Nordic welfare regimes–and contrast with those of Canada and England–market-oriented welfare regimes [21]. However, the likelihood of employment in Denmark was closer to the Canadian results than the Swedish results. Larsen and Pedersen [44] also found differences in employment between Denmark and Sweden for this age group. Both are Nordic welfare states, known for universal social protection, but in recent years, Danish policy reforms have moved more towards inequity [45], sparking debate about whether Denmark is still a Nordic welfare state [46, 47]. Furthermore, in Denmark, no legal protection exists to prevent employers from dismissing employees as a result of their illness and while on sick leave, unless it is obvious discrimination, potentially leaving persons with ill-health more vulnerable to unemployment or more in need of using early-retirement schemes, despite having otherwise strong employment protection regulations. Denmark’s early-retirement schemes could also be another possible explanation of why odds of employment are lower in Denmark than Sweden. In Sweden, on the other hand, employment protection policies are more pronounced and might explain the higher likelihood of employment among older persons with health conditions. Such differences could lead to lower probability of employment for persons with poor health in Denmark compared to Sweden. Differences in pension age could be another explanation. For example, substantially lower pension eligibility ages for English women could partially explain the lower chances of employment for English women compared with Canadian women [48], although this is changing. It should also be noted that none of the comparison countries have mandatory retirement laws enacted at the time of this study (or during the years of data collection).

### Limitations

As with most comparative studies, challenges arise in harmonising variables across datasets. In particular, mental health measures are rarely consistent across settings. A further shortcoming of the study is the modest size of the SHARE surveys and the relatively high non-response rates might introduce a bias [49] because the distribution of educational level among the participants does not reflect that of the population. The low sample sizes allowed only a dichotomization of level of education and also demonstrate a need for better employment data on older persons aged 65 years and older. We also need more longitudinal studies about employment trajectories at older ages.

Furthermore, there are of course more physical illnesses beyond the five that were selected which could affect employment, but we chose to focus on these five, given that these have a large burden of disease in high-income settings in this age group, as well as for methodological convenience. The same applies to the mental health measure which was limited to depression as this was the only mental health measure available for cross-country comparisons. Nevertheless, any omitted physical or mental conditions would have been placed in the comparison group, and as such we have if anything underestimated the strength of the associations between health and employment. We also recognize that our threshold of 15 hours per week employment could impact analysis and not capture those that work less than 15 hours per week.

## Conclusion

The study has revealed certain differences between the four countries and between population groups, with respect to the degree to which gender, level of education and different health conditions and combinations of comorbidities correlate with the chances of employment among older persons. Our results suggest that groups with low education and physical-mental health comorbidity are especially vulnerable in this respect. Further studies are warranted, with larger sample sizes allowing more detailed analyses to guide policy. Meanwhile, given the between-country differences observed, future in-depth comparative studies of country-level policies to facilitate extending working lives could contribute to ways of increasing the employment rates of persons beyond retirement age such as natural experiment studies. In light of this study, we suggest that policy-makers consider sub-groups when designing policies to extend working lives as a ‘one size fits all’ approach may work for some groups, whilst leaving vulnerable groups behind, thus perpetuating health inequalities at older age.
